# Low-Quality Housing Is Associated With Increased Risk of Malaria Infection: A National Population-Based Study From the Low Transmission Setting of Swaziland

**DOI:** 10.1093/ofid/ofx071

**Published:** 2017-04-06

**Authors:** Nomcebo Dlamini, Michelle S. Hsiang, Nyasatu Ntshalintshali, Deepa Pindolia, Regan Allen, Nomcebo Nhlabathi, Joseph Novotny, Mi-Suk Kang Dufour, Alemayehu Midekisa, Roly Gosling, Arnaud LeMenach, Justin Cohen, Grant Dorsey, Bryan Greenhouse, Simon Kunene

**Affiliations:** 1 Swaziland National Malaria Control Programme, Manzini; 2 Department of Pediatrics, University of Texas Southwestern Medical Center, Dallas; 3 Malaria Elimination Initiative, Global Health Group, and Departments of; 4 Pediatrics and; 5 Medicine, University of California San Francisco; and; 6 Clinton Health Access Initiative, Boston, Massachusetts

**Keywords:** housing, low transmission, malaria elimination, vector control, Swaziland

## Abstract

**Background:**

Low-quality housing may confer risk of malaria infection, but evidence in low transmission settings is limited.

**Methods:**

To examine the relationship between individual level housing quality and locally acquired infection in children and adults, a population-based cross-sectional analysis was performed using existing surveillance data from the low transmission setting of Swaziland. From 2012 to 2015, cases were identified through standard diagnostics in health facilities and by loop-mediated isothermal amplification in active surveillance, with uninfected subjects being household members and neighbors. Housing was visually assessed in a home visit and then classified as low, high, or medium quality, based on housing components being traditional, modern, or both, respectively.

**Results:**

Overall, 11426 individuals were included in the study: 10960 uninfected and 466 infected (301 symptomatic and 165 asymptomatic). Six percent resided in low-quality houses, 26% in medium-quality houses, and 68% in high-quality houses. In adjusted models, low- and medium-quality construction was associated with increased risk of malaria compared with high-quality construction (adjusted odds ratio [AOR], 2.11 and 95% confidence interval [CI], 1.26–3.53 for low vs high; AOR, 1.56 and 95% CI, 1.15–2.11 for medium vs high). The relationship was independent of vector control, which also conferred a protective effect (AOR, 0.67; 95% CI, .50–.90) for sleeping under an insecticide-treated bed net or a sprayed structure compared with neither.

**Conclusions:**

Our study adds to the limited literature on housing quality and malaria risk from low transmission settings. Housing improvements may offer an attractive and sustainable additional strategy to support countries in malaria elimination.

Malaria control and elimination strategies target the parasite reservoir in humans, through case management and surveillance, or in mosquitoes, via vector control. In Africa, where malaria vectors feed primarily indoors at night, vector control has generally been achieved through wide-scale delivery of insecticide-treated bed nets (ITNs) and indoor residual spraying (IRS), the spraying of insecticide on the interior walls of homes [1–3]. Of the 40% decrease in incidence of malaria clinical disease between 2000 and 2015 in Africa, 68% is attributed to ITNs and 10% to IRS [4]. Because ITNs require replacement every 3 to 5 years, and IRS is performed at least once yearly, these interventions generally consume one quarter to more than half the budget of a malaria program. With limited domestic and donor funding for malaria, particularly in low transmission settings where the perceived threat of malaria is the lower, continued costs are a challenge [5, 6]. Furthermore, high coverage and acceptability are challenging [7], and even with high coverage of additional interventions such as mass testing and treatment, elimination may not be achievable [8]. The threat of insecticide resistance is an additional impetus to find alternative and sustainable strategies.

In the early 20th century, malaria control efforts included housing modifications such as covering windows with mesh and constructing screened porches around front doors [9, 10]. Despite resulting vast declines in malaria transmission worldwide, including in the United States, Italy, South Africa, Panama, and India, the strategy is uncommonly used today [11–14]. With the development of dichlorodiphenyltrichloroethane (DDT) in the 1950s, indoor house spraying became the mainstay for malaria elimination efforts and housing modification fell out of favor. A recent meta-analysis found significantly lower odds of malaria infection and disease in residents of modern compared with traditional houses [15]. Overall housing quality as well as specific housing components (eg, door and windows screens, closed eaves, brick walls, and tiled or metal roofs) were associated with reduced mosquito entry and/or improved clinical outcomes. However, it was limited by the overall low quality of available studies, and few studies from very low transmission settings, where housing quality may be less relevant to malaria risk due to other drivers such as occupation and behavioral factors [16]. Further evidence on the association between housing (overall quality as well as quality of individual components) and malaria risk in low transmission settings may support improved housing as an additional tool to reach elimination.

The objective of this study was to evaluate the association between housing quality and locally acquired infection in the low transmission setting of Swaziland. Due to effective case management, surveillance and response, and greater use of long-lasting ITNs (LLINs) and IRS, incidence from 1999 to 2009 fell from 3.9 to 0.07 cases per 1000 [17, 18]. However, incidence has since plateaued. To achieve and sustain elimination, additional strategies may be necessary. Specific study aims were to (1) measure the association between overall housing quality and malaria risk, (2) measure the associations between individual housing components and malaria risk, and (3) determine whether any protection provided by housing quality was independent of that provided by standard vector control. We anticipated that findings would inform malaria elimination planning in Swaziland and other low transmission settings.

## METHODS

### Study Design

A population-based cross-sectional analysis was conducted using existing national data collected from passive and active surveillance from August 2012 to March 2015.

### Setting

Swaziland is a low-middle income country in southern Africa. It is considered a very low transmission setting with parasite prevalence last measured at 0.2% in 2010 [19]. Malaria transmission is seasonal and occurs in the eastern rural, agricultural areas. Plasmodium *falciparum* is the primary species, and the principal vector, *Anopheles arabiensis*, is indoor biting and resting. Approximately half of passively identified cases are classified as imported, mostly from Mozambique, which borders the country on the east [20]. Swaziland currently has 261 public and private health facilities, all of which report newly confirmed cases of malaria to the Ministry of Health’s immediate disease notification system.

### Study Population

The study population consisted of children and adults residing in malaria-endemic regions of Swaziland who were identified through either passive or active surveillance. Cases included subjects with confirmed malaria infection. Passively detected cases were symptomatic and diagnosed by *P falciparum*-specific RDT (rapid diagnostic test) and/or microscopy at a health facility and reported to the national disease notification system. Actively detected cases included largely asymptomatic loop-mediated isothermal amplification (LAMP)-detectable infections found through reactive case detection (RACD), a strategy of testing household members and neighbors of index cases to find additional malaria cases [21]. Uninfected subjects from RACD served as a comparison group. Residence in the eastern endemic area of the country was an inclusion criteron. Exclusion criteria included the following: refusal to participate, travel in the prior 8 weeks but excluding the last week, residence in an institutional home (eg, military base), or lack of geographic (geo)-coordinates of residence for index cases.

### Data Collection

Survey data were collected by surveillance team members during home visits. After an index case was reported, surveillance agents attempted to visit their home to record geo-coordinates and conduct a questionnaire with the index case, household members, and neighbors. The questionnaire collected the following: demographics; detailed information about potential risk factors for malaria such as age, gender, occupation, travel history, and coverage and usage of vector control interventions; and fever history in RACD subjects. Interviews were conducted in the local languages, or Portuguese with Mozambicans. Housing quality was assessed by visual inspection.

The team aimed to interview the index case within 48 hours of the case report, and if the index case resided in the eastern endemic region, and RACD was not already conducted in the prior 5 weeks, RACD was initiated within 1 week. Household members and neighbors residing within 500 meters of index cases were targeted for *P falciparum*-specific RDT testing. Due to limitations of RDT to detect low-density asymptomatic infections [22], a dried blood spot was also collected for subsequent testing using LAMP, a molecular and more sensitive detection method. The RDT-positive subjects identified during RACD were transported to the nearest health facility for treatment. Ethics approval was granted by the Swaziland Ethics Committee and the Committee on Human Research at the University of California, San Francisco.

To account for potential ecological confounders of malaria risk, values for satellite-derived data were collected according to date of encounter and geo-coordinates. These included (1) normalized difference water index (NDWI) and (2) land surface temperature (LST) representing the average value during the prior 3 months and from the surrounding 500-meter radius from the Moderate Resolution Imaging Spectroradiometric instrument. Elevation data were collected from the Shuttle Radar Topography Mission.

### Laboratory Methods

Microscopy was conducted according to national guidelines, and RDT testing was performed using the First Response Malaria Ag *P falciparum* HRP-2 RDT (Premier Medical Corporation Ltd). Dried blood spots were generated using Whatman 3MM paper and transferred to 4°C within 1 week and −20°C within 1 month. A 6-mm punch was used for chelex extraction [23], 5 µL of which was used for *Plasmodium* genus-specific LAMP testing. If positive, an additional 5 µL was used for confirmatory *P falciparum*-specific LAMP testing (Loopamp Malaria Pan and Pf Detection Kits; Eiken Chemical Co., Ltd).

### Housing Typology

**Figure 1.**
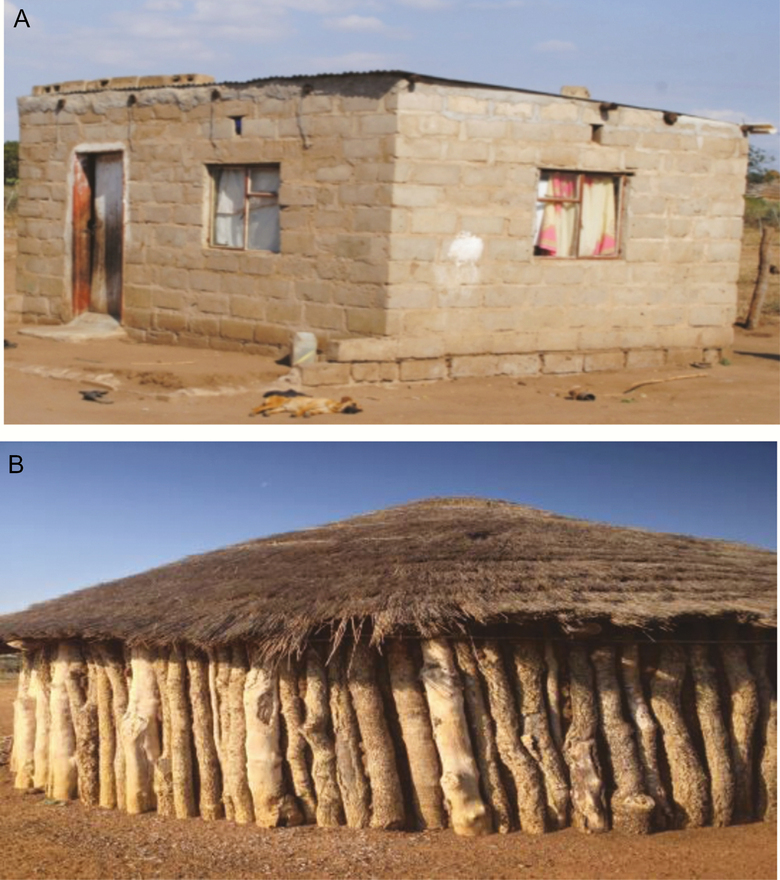
Examples of high- and low-quality homes. (A) High-quality housing with cement block internal and external walls, some screened windows, and metal roofing. (B) Low-quality housing with stick walls, grass roofing, and no windows.

Housing components were grouped into categories according to potential for mosquito entry and resting (Table 1). Because prior studies have found increased mosquito entry and resting with traditional compared with modern household construction materials [15], external wall, internal wall, and roof were classified as low quality if they were composed of natural materials and classified as high quality if composed of modern materials. Having no windows or unscreened windows was considered low quality due to the likelihood that the door would be kept open to maintain ventilation and thus enable mosquito entry. A composite housing index was also created according to a 3-category variable (low, medium, and high) that considered the 4 housing components (Table 1 and Figure 1). Houses were considered low quality if all 4 housing components were categorized as low quality. High-quality houses were constructed with 4 high-quality components. Medium-quality houses were classified as any house not otherwise fitting low- or high-quality criteria.

**Table 1.** Housing Component and Composite Model Criteria

	Individual Housing Component			
Quality of Overall Housing or Individual Component	External Wall	Internal Wall	Roof	Windows
High	Cement block, brick, plywood or wood	Cement block, brick, plywood, plaster, or wood	Metal sheets or tile	Screened window
Medium^a^	Any house not otherwise fitting low- or high-quality criteria			
Low	Cane, grass, shrub, or mud (includes stick and mud)	Cane, grass, shrub, or mud (includes stick and mud)	Grass or palm	No window or unscreened window

^a^Only applies to overall housing quality (composite model).

### Data Management and Analysis

Data were collected electronically on tablets and cleaned and analyzed in Stata 13.0 (StataCorp). For all analyses, the outcome variable was malaria, defined by a positive RDT and/or microscopy result for symptomatic index cases, and LAMP positivity in RACD-identified cases. As described above, the primary exposure of interest was residence in a low- or medium-quality house compared with a high-quality house. Secondary exposures of interest were residence in a house with individual housing components of low or high quality.

Initial analyses showed certain occupations to confer higher odds of malaria infection. Higher risk occupations included farming, manufacturing, other manual labor, and small-market sales or trade, whereas low-risk occupations included office work, student, unemployed, and other. A binary variable for the covariate of occupation (high relative to low risk) was subsequently generated. Bed net and IRS use were evaluated separately and collapsed in the bivariate analysis, with the latter included in the adjusted analysis because one goal of the analysis was to evaluate the associate of housing quality independent from other any other vector control intervention. Furthermore, there was little overlap of ITN and IRS use. Although 33.9% used one or the other, only 7.9% used both. Therefore, these 2 categories were collapsed into a single category in a binary variable of any vector control relative to none.

Bivariate logistic regression models were used to assess relationships between covariates and locally acquired infection. Multivariate logistic regression models were then used for the 2 primary predictor variables: individual housing component and overall housing quality. Although the primary aim of the study was to evaluate overall housing quality, the analysis of individual components was also included should malaria risk be mainly associated with 1 or a few housing components, in which case housing improvement interventions could be streamlined. Covariates were included in the multivariate analysis if the relationship in the bivariate analysis was significant (95% confidence interval [CI] not including 1.0). Vector control coverage was included in the multivariate analysis as a collapsed variable because a goal of the analysis was to evaluate the association of housing quality to malaria risk, independent of any other vector control intervention. Due to collinearity between some variables, the most representative variable was used (eg, the collapsed vector control coverage variable) or Akaike information criterion was used to select the model of best fit (LST compared with NDWI, and internal compared with external wall). To test whether selection of cases from different sources introduced bias, an analysis that was restricted to subjects from RACD (excluding index cases from health facilities) was performed. In all analyses, a household level random effect was included to account for household-level clustering.

## RESULTS

### Characteristics of the Study Population

**Figure 2.**
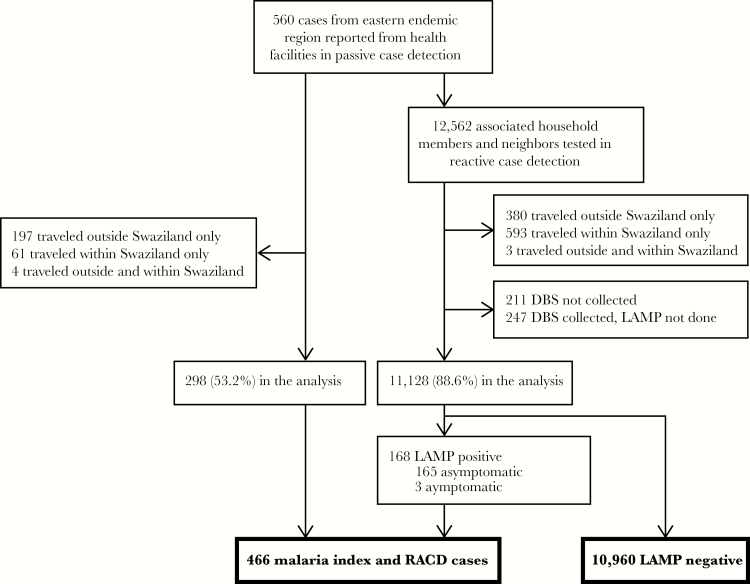
Study subject inclusion process with infection data. DBS, dried blood sample; LAMP, loop-mediated isothermal amplification; RACD, reactive case detection.

In the analysis, 298 (53.2%) subjects were included due to report of no travel (Figure 2). In RACD, 11128 (88.6%) of 12562 subjects met inclusion criteria. Subjects lived in homes with mostly high-quality individual housing components. Proportion of subjects residing in a home with individual components of low quality were 17.4% for external wall, 15.4% for internal wall, 10.8% for roof, and 24.0% for window. Overall housing quality was high in 67.8%, medium in 26.1%, and low in 6.2%. Study subjects were mostly under 40 years (80.3%), Swazi nationality (99.1%), and female (57.0%). The majority of participants (89.7%) held occupations with lower risk of malaria infection. A total of 10.3% of subjects held higher risk occupations. Household LLIN ownership was reported in 22.2%, 13.2% reported sleeping under an LLIN the night prior, and 36.2% slept under an insecticide-sprayed structure the night prior (Table 2). Taken together, 41.8% slept under an LLIN and/or sprayed structure and 58.2% were protected by neither. Among RACD subjects, 9.2% resided in the index case household and 78.8% resided in neighboring households within 500 meters. Twelve percent resided beyond the goal 500 meters but were included in the analysis due to their proximity to the index case (median distance 633 meters in this group).

**Table 2.** Associations Between Demographic, Environmental, and Housing Characteristics and Malaria Infection

Predictor Variable		Total (%)	No. Infected (%)	Unadjusted OR (95% CI)	Housing Components Model AOR (95% CI)	Overall Housing Model AOR (95% CI)
Age (years)	<15	5042 (44.1)	179 (3.6)	1	1	1
	15–40	4130 (36.2)	214 (5.2)	2.13 (1.63–2.79)	1.81 (1.32–2.48)	1.80 (1.31– 2.47)
	≥40	2254 (19.7)	73 (3.2)	1.91 (1.38–2.67)	1.68 (1.15–2.47)	1.67 (1.14–2.46)
Gender	Female	6508 (57.0)	176 (2.7)	1	1	1
	Male	4918 (43.0)	290 (5.9)	1.98 (1.55–2.52)	1.90 (1.45–2.49)	1.89 (1.45–2.48)
Nationality	Swazi	11,319 (99.1)	449 (4.0)	1	1	1
	Mozambican	70 (0.6)	13 (18.6)	5.69 (2.42–13.37)	4.41 (1.79–10.84)	4.47 (1.84–10.87)
	South African	3 (0.0)	0 (0.0)	^e^	^e^	^e^
	Other^a^	34 (0.3)	4 (11.8)	4.05 (1.36–12.10)	2.86 (0.82–10.01)	2.49 (0.71–8.78)
Occupation^b^	Lower risk^b^	10 193 (89.7)	381 (3.7)	1	1	1
	Higher risk^b^	1174 (10.3)	80 (6.8)	2.81 (2.08–3.79)	2.11 (1.50–2.96)	2.11 (1.50–2.98)
Malaria season	Low	4631 (40.5)	238 (5.1)	1	-	-
	High	6795 (59.5)	228 (3.4)	0.80 (0.60–1.06)	-	-
Transmission year (September to June)	2012–2013	1731 (15.2)	69 (4.0)	1	-	-
	2013–2014	5784 (50.6)	179 (3.1)	0.77 (0.48–1.23)	-	-
	2014–2015	3911 (34.2)	218 (5.6)	1.45 (0.90–2.34)	-	-
Water body distance (m)^c^	>1000	3056 (29.9)	131 (4.3)	1	-	-
	101–1000	3323 (32.6)	145 (4.4)	1.05 (0.73–1.50)	-	-
	≤100	3830 (37.5)	169 (4.4)	1.19 (0.83–1.70)	-	-
Ecological covariates, mean (SD)^c^	LST (°C)	29.5 (3.3)	28.9 (3.0)	0.95 (0.91–0.98)	0.95 (0.92–0.99)	0.95 (0.91–0.99)
	NDWI (−1 to 1)	−0.07 (0.07)	−0.08 (0.07)	0.18 (0.02–1.80)	-	-
	Elevation (10 meters)	35.3 (15.8)	34.8 (14.2)	0.99 (0.99–1.00)	-	-
Vector control coverage						
Slept outside in the past week	No	11 298 (98.9)	456 (4.0)	1	-	-
	Yes	128 (1.1)	10 (7.8)	2.71 (1.30–5.67)	-	-
Household bed net ownership	No	8890 (77.8)	367 (4.1)	1	-	-
	Yes	2536 (22.2)	99 (3.9)	0.71 (0.50–1.00)	-	-
Slept under bed net in the past week	No	9915 (86.8)	427 (4.3)	1	-	-
	Yes	1511 (13.2)	39 (2.6)	0.47 (0.29–0.76)	-	-
Slept under structure sprayed in past year^c^	No	6883 (63.8)	332 (4.8)	1	-	-
	Yes	3908 (36.2)	109 (2.8)	0.67 (0.49–0.91)	-	-
Slept under bed net in the past week and/or structure sprayed in the past year^c^	No	6336 (58.2)	309 (4.9)	1	1	1
	Yes	4554 (41.8)	134 (2.9)	0.65 (0.48–0.87)	0.66 (0.49–0.89)	0.67 (0.50–0.90)
Housing components^d^						
External wall^c^	High-quality	9405 (82.6)	348 (3.7)	1	1	-
	Low-quality	1980 (17.4)	114 (5.8)	1.74 (1.27–2.38)	1.54 (0.94–2.53)	-
Internal wall^c^	High-quality	9595 (84.6)	362 (3.8)	1	-	-
	Low-quality	1748 (15.4)	99 (5.7)	1.62 (1.16–2.26)	-	-
Roof^c^	High-quality	10 123 (89.3)	392 (3.9)	1	1	-
	Low-quality	1219 (10.8)	72 (5.9)	1.55 (1.07–2.25)	1.01 (0.60–1.71)	-
Windows	High-quality	8680 (76.0)	321 (3.7)	1	1	-
	Low-quality	2746 (24.0)	145 (5.3)	1.46 (1.10–1.93)	1.26 (0.89–1.77)	-
Overall housing quality^c,d^	High-quality	7632 (67.8)	261 (3.4)	1	-	1
	Medium-quality	2936 (26.1)	152 (5.2)	1.57 (1.18–2.08)	-	1.56 (1.15–2.11)
	Low-quality	693 (6.2)	46 (6.6)	2.13 (1.34–3.40)	-	2.11 (1.26–3.53)

Abbreviations: AOR, adjusted odds ratio; CI, confidence interval; LST, land surface temperature; NDWI, normalized difference water index; OR, odds ratio; SD, standard deviation.

^a^There were 34 subjects of other nationalities, and Burundi (*n* = 10) and Zimbabwean (*n* = 10) were most highly represented.

^b^Higher risk occupations included the following: farming, manufacturing, other manual labor, and small-market sales or trade. Lower risk occupations included office work, student, unemployed, and other.

^c^n(uninfected), n(infected): occupation: 10 906, 461; water body distance: 9764, 445; ecological covariates: 446, 10 885; slept under sprayed structure: 10 350, 441; slept under bed net in the past week and/or structure sprayed in the past year: 10 447, 443; external wall: 10 923, 462; internal wall: 10 882, 461; roof: 10 878, 464; overall housing quality: 10 802, 459.

^d^External wall: high-quality (plywood or wood, cement block, or brick), low-quality (cane, grass, shrub, or mud, including stick and mud); internal wall: high-quality (plaster, plywood, or wood, cement block, or brick), low-quality (cane, grass, shrub, or mud, including stick and mud); roof: high-quality (metal sheets or tile), low-quality (grass or palm); windows: high-quality (screened window), low-quality (no window or unscreened window); overall housing quality: high-quality (high-quality external wall, internal wall, roof and windows), low-quality (low-quality external wall, internal wall, roof, and windows), medium-quality (not otherwise fitting low- or high-quality criteria).

^e^Predicted perfectly.

### Infection Data

There were 10960 LAMP-negative subjects (10232 RDT negative, 6 RDT positive, and 722 RDT not done due to an RDT stock-out). Of 168 LAMP-positive RACD-identified cases, 115, 48, and 5 were RDT negative, positive, and not done, respectively, and 165 were asymptomatic and 3 were symptomatic. Subpatent, or low-density infections missed by RDT, represented 71.9% of asymptomatic cases and 24.7% of all RACD-identified cases.

Of 2535 total households, 232 (9.2%) had malaria cases, 14.2% (*n* = 33) of which had more than 1 malaria case. Of the 168 RACD-identified cases, 34 (20.2%) resided in the same household as one of the 26 index cases, and all but 2 of these index cases were considered to have locally acquired infection.

### Associations Between Demographic, Behavioral, and Ecological Factors With Infection

Age ≥15 years, male gender, nationality other than Swazi, higher risk occupations, lower land surface temperature, and lower NDWI were associated with higher odds of infection (Table 2). The relationships with season, transmission year, distance to a water body, and elevation were not statistically significant. In the multivariate analyses, the relationships with age, gender, Mozambican nationality, occupation, and LST remained significant.

### Vector Control Coverage

Not sleeping outside, LLIN ownership, sleeping under an LLIN, and sleeping under a sprayed structure were associated with lower unadjusted odds of infection. In the multivariate analysis, sleeping under an LLIN and/or a sprayed structure, compared with neither, remained protective in both models: AOR 0.66, 95% CI 0.49–0.89, and AOR 0.67, 95% CI 0.50–0.90, respectively.

### Relationship Between Housing Quality and Malaria Infection

In the multivariate model, there were trends in the associations between low-quality individual housing components and infection, but the relationships were not significant (Table 2). With the composite housing quality model, medium- and low-quality housing were associated with infection in the bivariate analysis and these relationships remained statistically significant in the multivariate models (adjusted odds ratio [AOR] 1.56 and 95% CI, 1.15–2.11 and AOR 2.11 and 95% CI, 1.26–3.53, respectively). In the analyses restricted to subjects from RACD, low-quality external wall, roof, and windows were each associated with 1.65 (95%, CI 0.82–3.31), 1.09 (95% CI, 0.55–2.19), and 1.42 (95% CI, 0.91–2.23) odds of infection, respectively. Compared with overall high-quality housing, medium- and low-quality housing were associated with 1.85 (95% CI, 1.24–2.75) and 2.68 (95% CI, 1.40–5.14) higher odds of infection, respectively.

## DISCUSSION

Recent studies have found that individuals living in traditional compared with modern homes have 2-fold higher odds of malaria infection, but the evidence has generally been of poor quality and limited to moderate and high transmission settings [15, 24]. In this 3-year national population-based study in the low transmission setting of Swaziland, we found that residence in a low- or medium-quality house, relative to a high-quality house, was an important determinant of locally acquired malaria infection. Even after adjusting for a comprehensive set of potential confounders, such as adult age, male gender, and certain occupations, which are known risk factors in low transmission settings [16], as well as lower LST, which is associated with higher moisture and thus mosquito breeding [25], and bed net and IRS coverage, we found a strong association between low-quality housing and infection.

Swaziland is among 35 countries that have been successful in malaria control using standard interventions, and achievement of elimination goals will require additional interventions that are effective, acceptable, and sustainable [26]. Housing improvements are thought to have contributed to malaria declines and elimination in many countries, but these remedies fell and remained out of favor when IRS and later ITNs became available [3, 11–14]. More recently, malaria elimination in the United Arab Emirates in 2007 has been attributed to provision of inexpensive screened or air-conditioned housing to the at-risk population of migrant workers [27], and housing improvement as part of economic development in Hainan Province, China may have contributed to its 2010 malaria elimination [28].

Our low transmission cited study adds critical information to the existing literature on housing quality and malaria risk. Low-quality housing likely enables mosquito entry into homes through holes or cracks in traditional materials such as stick, mud, and grass. Modern materials such as wood, cement blocks, or metal are less permeable to vector entry. It is also hypothesized that roofs made of metal, compared with grass or palm, trap heat and discourage mosquito resting. Unscreened windows, the most common low-quality housing component in our study, likely facilitate mosquito entry, with lack of windows encouraging mosquito entry through doors left open for ventilation. It is interesting to note that low-quality walls, roofs, and windows were each associated with infection in the unadjusted analysis, but the relationship was not as significant in the adjusted analysis as it was for the overall housing quality model. Because the composite housing outcome was constructed as an ordinal variable according to specific housing components (Table 1), the finding suggests that there may be a dose-dependent or synergistic effect among low-quality housing components that promotes mosquito entry and/or resting. As such, housing improvements that are holistic rather than focused on specific components may be more effective.

The finding that farming, manual labor, and small-market sales, the latter which often takes place in outdoor stands, suggests that malaria is acquired from outdoor biting mosquitoes. Data from other low transmission settings seem to support such a change in mosquito behavior [29]. In Swaziland, the primary vector is *A arabiensis*. This highly adaptable mosquito, perhaps due to environmental factors (eg, land/water use changes), may be encouraged to feed outdoors where it is less vulnerable to indoor preventative interventions such as IRS and ITNs. Because this behavior could also work against the desired impact of housing improvement, ongoing vector surveillance should be maintained.

It should be noted that cases were from 2 different sources, potentially introducing selection bias. However, in the analysis restricted to RACD-identified cases and uninfected subjects from RACD, there was an even higher AOR of 2.78, compared with 2.11 in the main analysis, suggesting that low-quality housing confers an even higher risk of infection in a population where local transmission is more likely (RACD was only implemented in villages with index cases). The nonrandom sampling of controls in the neighborhoods of index cases could be considered a potential limitation. However, the controls were representative of the population of interest, or those at highest risk of malaria infection. In this study, prevalence of infection among subjects residing near index cases was 1.5% in this study, relative to 0.2% in randomly sampled subjects in a previous national cross-sectional survey [19]).

We recognize other potential limitations of the study. First, we did not collect information on holes, cracks, open doorways, or eaves [30], and therefore we could not directly measure their associations with infection. Future evaluations should include such details. Second, it could be suggested that a more direct outcome measure of mosquito entry, such as mosquito density, should have been used. However, we relied on routine surveillance data, and such detailed assessments in almost 3000 households may not have been feasible. Finally, observational studies are limited in their ability to assess causality. For example, low-quality housing may represent lower education or socioeconomic status, which we did not directly assess, but, in the past, we have not found it to be a risk factor within the eastern endemic region, which is largely rural [19]. A randomized controlled trial would be the gold standard study design [31]. For some countries that need evidence to support elimination by 2020 (the goal for most of the 35 eliminating countries) [26], it may not be practical to implement such a resource- and time-intensive exercise, nor is it feasible to measure impact when the malaria incidence is already low. However, modified interventions studies, such as before and after assessments, or phased implementation evaluations in higher risk areas should be pursued.

There are several strengths of our study. First, we used a large and robust national dataset covering 3 transmission seasons. We included symptomatic and asymptomatic infections, the latter constituting 35% of cases. We used a highly sensitive diagnostic to measure subpatent infections, which represented one quarter of all infections and 72% of asymptomatic infections. Subpatent infections represent an increasing proportion of infections in low transmission settings [22], and our knowledge has not been included in previous published studies on housing and infection [15].

## CONCLUSIONS

Our study adds to the limited literature on housing quality and malaria risk from low transmission settings. We found that living in overall low-quality housing was associated with increased risk of malaria infection. Further studies demonstrating the causal relationship between specific housing attributes and lower malaria risk could help inform the design of housing modifications. Housing improvements may offer an attractive and sustainable additional strategy to support countries in malaria elimination.

## References

[1] Sinka ME Bangs MJ Manguin S et al. A global map of dominant malaria vectors. Parasit Vectors 2012; 5:69. 22475528 10.1186/1756-3305-5-69 PMC3349467

[2] Gillies MT de Meillon B. *The Anophelini of Africa South of the Sahara (Ethiopian Zoogeographical Region*). 2nd ed Johannesburg: South African Institute for Medical Research; 1968.

[3] World Health Organization. World Malaria Report 2015. Geneva: World Health Organization; 2015.

[4] Bhatt S Weiss DJ Cameron E et al. The effect of malaria control on *Plasmodium falciparum* in Africa between 2000 and 2015. Nature 2015; 526:207–11. 26375008 10.1038/nature15535 PMC4820050

[5] Sabot O Cohen JM Hsiang MS et al. Costs and financial feasibility of malaria elimination. Lancet 2010; 376:1604–15. 21035839 10.1016/S0140-6736(10)61355-4 PMC3044845

[6] Mullan Z Malaria: can we mention the e-word yet? Lancet Glob Health 2016; 4:e344. 27121761 10.1016/S2214-109X(16)30068-7

[7] Rehman AM Coleman M Schwabe C et al. How much does malaria vector control quality matter: the epidemiological impact of holed nets and inadequate indoor residual spraying. PLoS One 2011; 6:e19205. 21559436 10.1371/journal.pone.0019205 PMC3084796

[8] Griffin JT Hollingsworth TD Okell LC et al. Reducing *Plasmodium falciparum* malaria transmission in Africa: a model-based evaluation of intervention strategies. PLoS Medicine 2010; 7:e1000324. 20711482 10.1371/journal.pmed.1000324 PMC2919425

[9] Lindsay SW Emerson PM Charlwood JD Reducing malaria by mosquito-proofing houses. Trends Parasitol. 2002; 18:510–4. 12473368 10.1016/s1471-4922(02)02382-6

[10] Lane CA. *Housing and Malaria: A Critical Summary of the Literature Dealing with the Subject*. League of Nations, 1931.

[11] Boyd MF The influence of obstacles unconsciously erected against anophelines (housing and screening) upon the incidence of malaria. Am J Trop Med 1926; 6:157–60.

[12] Celli A. *The New Preventative Treatment of Malaria in Latium. Collected Papers on Malaria. Angelo Celli, 1899–1912*. London: London School of Hygiene and Tropical Medicine; 1901.

[13] Le Prince JA Orenstein AJ *Mosquito Control in Panama; the Eradication of Malaria and Yellow Fever in Cuba and Panama*. London: G.P. Putnam’s Sons; 1916.

[14] Ross R Malaria prevention in Greece. BMJ 1913; 1:1186.

[15] Tusting LS Ippolito MM Willey BA, et al The evidence for improving housing to reduce malaria: a systematic review and meta-analysis. Malar J 2015; 14:209. 26055986 10.1186/s12936-015-0724-1 PMC4460721

[16] Cotter C Sturrock HJ Hsiang MS et al. The changing epidemiology of malaria elimination: new strategies for new challenges. Lancet 2013; 382:900–11. 23594387 10.1016/S0140-6736(13)60310-4 PMC10583787

[17] Sharp BL Kleinschmidt I Streat E et al. Seven years of regional malaria control collaboration–Mozambique, South Africa, and Swaziland. Am J Trop Med Hyg 2007; 76:42–7. 17255227 PMC3749812

[18] Kunene S Phillips AA Gosling RD et al. A national policy for malaria elimination in Swaziland: a first for sub-Saharan Africa. Malar J 2011; 10. 10.1186/1475-2875-10-313 PMC3219738 22018266

[19] Hsiang MS Hwang J Kunene S et al. Surveillance for malaria elimination in Swaziland: a national cross-sectional study using pooled PCR and serology. PLoS One 2012; 7:e29550. 22238621 10.1371/journal.pone.0029550 PMC3253098

[20] 2014–2015 Annual Malaria Report. Mbabane: National Malaria Control Programme, Ministry of Health, 2015.

[21] Sturrock HJ Hsiang MS Cohen JM et al. Targeting asymptomatic malaria infections: active surveillance in control and elimination. PLoS Med 2013; 10:e1001467. 23853551 10.1371/journal.pmed.1001467 PMC3708701

[22] Okell LC Bousema T Griffin JT et al. Factors determining the occurrence of submicroscopic malaria infections and their relevance for control. Nat Commun 2012; 3:1237. 23212366 10.1038/ncomms2241 PMC3535331

[23] Plowe CV Djimde A Bouare M et al. Pyrimethamine and proguanil resistance-conferring mutations in *Plasmodium falciparum* dihydrofolate reductase: polymerase chain reaction methods for surveillance in Africa. Am J Trop Med Hyg 1995; 52:565–8. 7611566 10.4269/ajtmh.1995.52.565

[24] Snyman K Mwangwa F Bigira V et al. Poor housing construction associated with increased malaria incidence in a cohort of young Ugandan children. Am J Trop Med Hyg 2015; 92:1207–13. 25870429 10.4269/ajtmh.14-0828 PMC4458827

[25] Midekisa A Senay G Henebry GM et al. Remote sensing-based time series models for malaria early warning in the highlands of Ethiopia. Malar J 2012; 11:165. 22583705 10.1186/1475-2875-11-165 PMC3493314

[26] Newby G Bennett A Larson E et al. The path to eradication: a progress report on the malaria-eliminating countries. Lancet 2016; 387:1775–84. 27116283 10.1016/S0140-6736(16)00230-0

[27] Sanders KC Rundi C Jelip J et al. Eliminating malaria in Malaysia: the role of partnerships between the public and commercial sectors in Sabah. Malar J 2014; 13:24. 24443824 10.1186/1475-2875-13-24 PMC3917703

[28] He CH Hu XM Wang GZ et al. Eliminating *Plasmodium falciparum* in Hainan, China: a study on the use of behavioural change communication intervention to promote malaria prevention in mountain worker populations. Malar J 2014; 13:273. 25017319 10.1186/1475-2875-13-273 PMC4112993

[29] Kibret S Wilson GG Increased outdoor biting tendency of *Anopheles arabiensis* and its challenge for malaria control in Central Ethiopia. Public Health 2016; 141:143–5. 27931990 10.1016/j.puhe.2016.09.012

[30] Knols BG Farenhorst M Andriessen R et al. Eave tubes for malaria control in Africa: an introduction. Malar J 2016; 15:404. 27515306 10.1186/s12936-016-1452-x PMC4982263

[31] Pinder M Conteh L Jeffries D et al. The RooPfs study to assess whether improved housing provides additional protection against clinical malaria over current best practice in The Gambia: study protocol for a randomized controlled study and ancillary studies. Trials 2016; 17:275. 27255167 10.1186/s13063-016-1400-7 PMC4891825

